# DBY-Tobacco: a dual-branch model for non-tobacco related materials detection based on hyperspectral feature fusion

**DOI:** 10.3389/fpls.2025.1538051

**Published:** 2025-03-05

**Authors:** Cheng Shen, Yuecheng Qi, Lijun Yun, Xu Zhang, Zaiqing Chen

**Affiliations:** ^1^ School of Information Science and Technology, Yunnan Normal University, Kunming, China; ^2^ Engineering Research Center of Computer Vision and Intelligent Control Technology, Department of Education of Yunnan Province, Kunming, China; ^3^ Yunnan Tobacco Leaf Company, Kunming, China

**Keywords:** object detection, non-tobacco related materials (NTRMs), hyperspectral imaging technology, feature fusion, deep learning

## Abstract

The removal of non-tobacco related materials (NTRMs) is crucial for improving tobacco product quality and consumer safety. Traditional NTRM detection methods are labor-intensive and inefficient. This study proposes a novel approach for real-time NTRM detection using hyperspectral imaging (HSI) and an enhanced YOLOv8 model, named Dual-branch-YOLO-Tobacco (DBY-Tobacco). We created a dataset of 1,000 images containing 4,203 NTRMs by using a hyperspectral camera, SpectraEye (SEL-24), with a spectral range of 400-900 nm. To improve processing efficiency of HSIs data, three characteristic wavelengths (580nm, 680nm, and 850nm) were extracted by analyzing the weighted coefficients of the principal components. Then the pseudo color image fusion and decorrelation contrast stretch methods were applied for image enhancement. The DBY-Tobacco model features a dual-branch backbone network and a BiFPN-Efficient-Lighting-Feature-Pyramid-Network (BELFPN) module for effective feature fusion. Experimental results demonstrate that the DBY-Tobacco model achieves high performance metrics, including an F1 score of 89.7%, mAP@50 of 92.8%, mAP@50-95 of 73.7%, and a processing speed of 151 FPS, making it suitable for real-time applications in dynamic production environments. The study highlights the potential of combining HSI with advanced deep learning techniques for improving tobacco product quality and safety. Future work will focus on addressing limitations such as stripe noise in HSI and expanding the detection to other types of NTRMs. The dataset and code are available at: https://github.com/Ikaros-sc/DBY-Tobacco.

## 1 Introduction

Tobacco leaves are the primary raw material for the production of cigarettes, cigars, and other tobacco products. Tobacco leaf farming and product production are important economic drivers in some countries and regions (Wang and Bennetzen, 2015). In recent years, China has become the largest tobacco producer and global cigarette market (Shahbandeh, 2024; Trenda, 2023). Tobacco products are among the most widely consumed goods globally. However, during the operations of tobacco leaf harvesting, shipping, and curing, other non-tobacco related materials (NTRMs) such as weeds, feathers, and rubber rings are frequently mixed in. NTRMs significantly reduce the quality of tobacco products and can endanger consumer health (Li et al., 2023). To be more specific, rubber products emit harmful substances during combustion and pyrolysis, which can have a negative impact on human health; materials like feathers and insect cocoons emit unpleasant odors when burned; and the presence of weeds and other foreign substances can affect the intrinsic quality of tobacco leaves.

The effective detection of NTRMs has long been a difficult problem. Currently, the primary method for removing NTRMs is manual screening, which is time-consuming and prone to subjectivity. Certain NTRMs are treated with specific ways, such as utilizing metal detectors to detect metal particles or air classifiers to remove lighter contaminants, but these approaches have limits.

Traditional detection and hyperspectral classification algorithms such as supervised Support Vector Machine (SVM) algorithms (El-Omairi et al., 2025), decision tree algorithms, and unsupervised approaches like K-means clustering (Wang et al., 2024) and PCA (Bagnasco et al., 2015) has been widely applied in various fields. However, with the development of computer vision, object detection methods based on deep learning are more popular. These methods have higher classification accuracy, especially in complex circumstances. Furthermore, the new technique is less susceptible to noise and has increased robustness (Spiegelberg and Rusz, 2017). Additionally, the scalability and generalization capability of these methods are impressive, allowing it to be applied to a variety of scenarios (El-Omairi et al., 2025).

Currently, the YOLO (You Only Look Once) algorithm is the most popular model for object detection (Jocher et al., 2023). Among the YOLO series, the most efficient algorithms in terms of performance are YOLOv5, YOLOv8, and YOLOv11. YOLOv5 adopts an Anchor-Based design, with anchor boxes to accommodate multi-scale and various aspect ratio targets. This design significantly enhances the model’s performance in dense object detection tasks. Additionally, YOLOv5 incorporates cross-scale skip connections within the network structure, enriching the gradient flow information. Building on YOLOv5, YOLOv8 introduces various improvements, with a primary focus on small object identification and computing resource optimization. The Anchor-Based design in YOLOv5 may waste computational resources when there are few targets, resulting in performance bottlenecks in small object identification tasks. Consequently, YOLOv8 discards the classic Anchor-Based design and proposes an Anchor-Free design, which is more suited to small object identification and simplifies the model. This innovation enables YOLOv8 to achieve greater precision in handling small objects while simplifying the network structure and enhancing model efficiency. YOLOv8 also incorporates more skip connections, which enriches the gradient flow and saves computing load via split operations. Moreover, YOLOv8’s decoupled head design separates the extraction and independent optimization of target location and class information, which improves the model’s robustness and generalization ability. The latest edition of the YOLO series, YOLOv11, incorporates cutting-edge technology innovations to improve object detection performance even more. YOLOv11 features modules designed to enhance the capability of feature extraction. These modules, through deep feature fusion and refined processing, effectively improve the model’s ability to capture detailed features. Additionally, YOLOv11 introduces attention mechanisms to further optimize feature extraction in critical regions. The attention mechanism allows the model to dynamically focus on crucial areas of an image while discarding unnecessary background information, enhancing object detection accuracy.

YOLO algorithm has been applied to various agricultural tasks in agricultural engineering, generating significant economic benefits (Badgujar et al., 2024; Furlanetto et al., 2024a, b; Zhou et al., 2025). Based on YOLOv5, Fan et al. (2024) achieved a lightweight improvement to the backbone by using ShuffleNet v2 (Ma et al., 2018), and enhanced the neck with BiFPN and a parallel hybrid attention mechanism (PHAM), allowing the model to better integrate multi-scale features. The improved model effectively identified weeds. Based on YOLOv7, Li et al. (2024) optimized the model’s backbone using the ConvNext module and combined the Swin Transformer (Liu et al., 2021) with the ConvNext module to improve the model’s head. This method achieved effective identification of foreign fibers in cotton while maintaining a high detection speed.

During training, the YOLO series object detection algorithms use manually annotated RGB images. However, RGB images only include spectral information from three predefined bands, resulting in limited data and minimal material distinction. RGB photos give insufficient spectrum information for advanced analysis tasks such as exact categorization or object detection. In recent years, hyperspectral imaging (HSI) has evolved as an accurate and non-destructive approach for target detection, solving RGB images’ shortcomings. This technology scans objects to obtain hundreds of wavelengths and extracts specific spectral information required for subsequent analysis, and it is widely used in agriculture to assess food quality (Du et al., 2020; Ram et al., 2024; Shuai et al., 2024). Zhang et al. (2024) combined Successive Projection Algorithm (SPA) with Principal Component Analysis (PCA) to select wavelengths of 1074 nm, 1269 nm, and 1441 nm for extracting bruise features from apples. Dong et al. (2014) selected wavelengths of 523 nm, 587 nm, 700 nm, and 768 nm through weighted coefficient analysis and PCA, effectively extracting the thrips defect features on Green-Peel citrus. It should be noted that these wavelengths are used as the characteristic wavelengths for the entire region (Dong et al., 2014; Vargas et al., 2006).

There has been increased attention to methods for feature fusion between HSIs and RGB images. To address the problem of performance degradation due to insufficient local feature interaction, Shen et al. (2024) proposed a feature fusion framework called Iterative Cross-Attention Guided Feature Fusion (ICAFusion). This framework improves object feature discriminability by using a query-guided cross-attention mechanism, which improves performance. To fully integrate different modalities, Qingyun et al. (2021) proposed a Cross-Modal Fusion Transformer (CFT) method. This approach uses the self-attention mechanism of Transformers to naturally perform both intra-modal and inter-modal fusion, dramatically improving multispectral object identification performance. Zhang et al. (2023) introduced SuperYOLO, an accurate and quick RSI object detection algorithm. This method integrates multimodal data and uses symmetric compact multimodal fusion (MF) to extract supplementary information from various data sources, resulting in better recognition of small objects.

There are some studies on NTRMs detection using computer vision. Li et al. (2023) proposed a method for classifying tobacco stems and impurities based on HSI superpixels and a Light Gradient Boosting Machine (LightGBM) classifier, achieving high accuracy. Wu et al. (2023) proposed an effective and rapid detection approach for NTRMs based on single-channel grayscale pictures and the YOLOv8 model. However, these studies are limited to detecting NTRMs in tobacco stems, and are ineffective for detecting NTRMs that are heavily hidden by tobacco leaves. Additionally, it is challenging to detect NTRMs that have colors similar to tobacco leaves. Furthermore, it is difficult to recognize NTRMs with hues comparable to tobacco leaves. As a result, this study performs spectrum analysis on NTRMs and improves the YOLOv8 algorithm to propose a model for effective and real-time NTRM detection.

The main works of this paper are the following: (1) Constructing the first HSIs dataset with 1,000 tobacco leaf images containing 4,203 NTRMs. (2) Using PCA to determine the characteristic wavelengths of NTRMs and pseudo color composition and decorrelation contrast stretch methods to generate images with significant NTRMs features. (3) Building on the YOLOv8n, an enhanced model, Dual-branch-YOLO-Tobacco (DBY-Tobacco), was proposed, which includes a dual-branch backbone network and a feature fusion module for the neck component.

**Figure 1:**
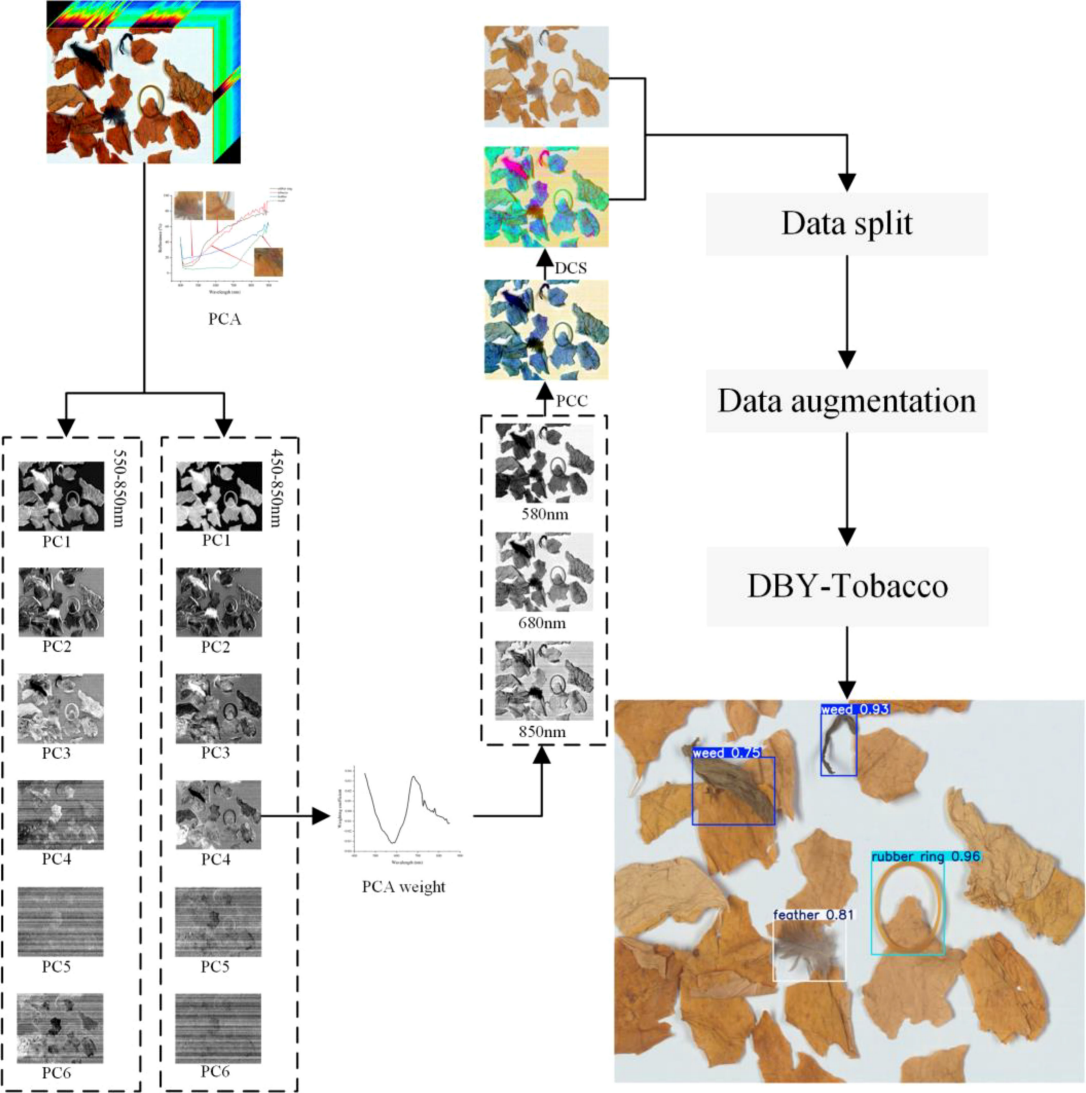
The overall technical route.

The overall technical route is shown in **Figure 1**. Firstly, we analyzed the reflectance of regions of interest (ROIs) in the hyperspectral images and identified two spectral regions where the reflectance differences among various NTRMs were most significant. PCA was then applied to these spectral regions to identify the Principal Component (PC) images that best describe the properties of various NTRMs. The weight coefficient map was used to produce three characteristic wavelengths. For image processing, techniques including pseudo color composition and decorrelation contrast stretch were used, and the processed images were subsequently input into the proposed model for real-time detection of NTRMs.

## 2 Materials and methods

### 2.1 Experimental environment

**Figure 2:**
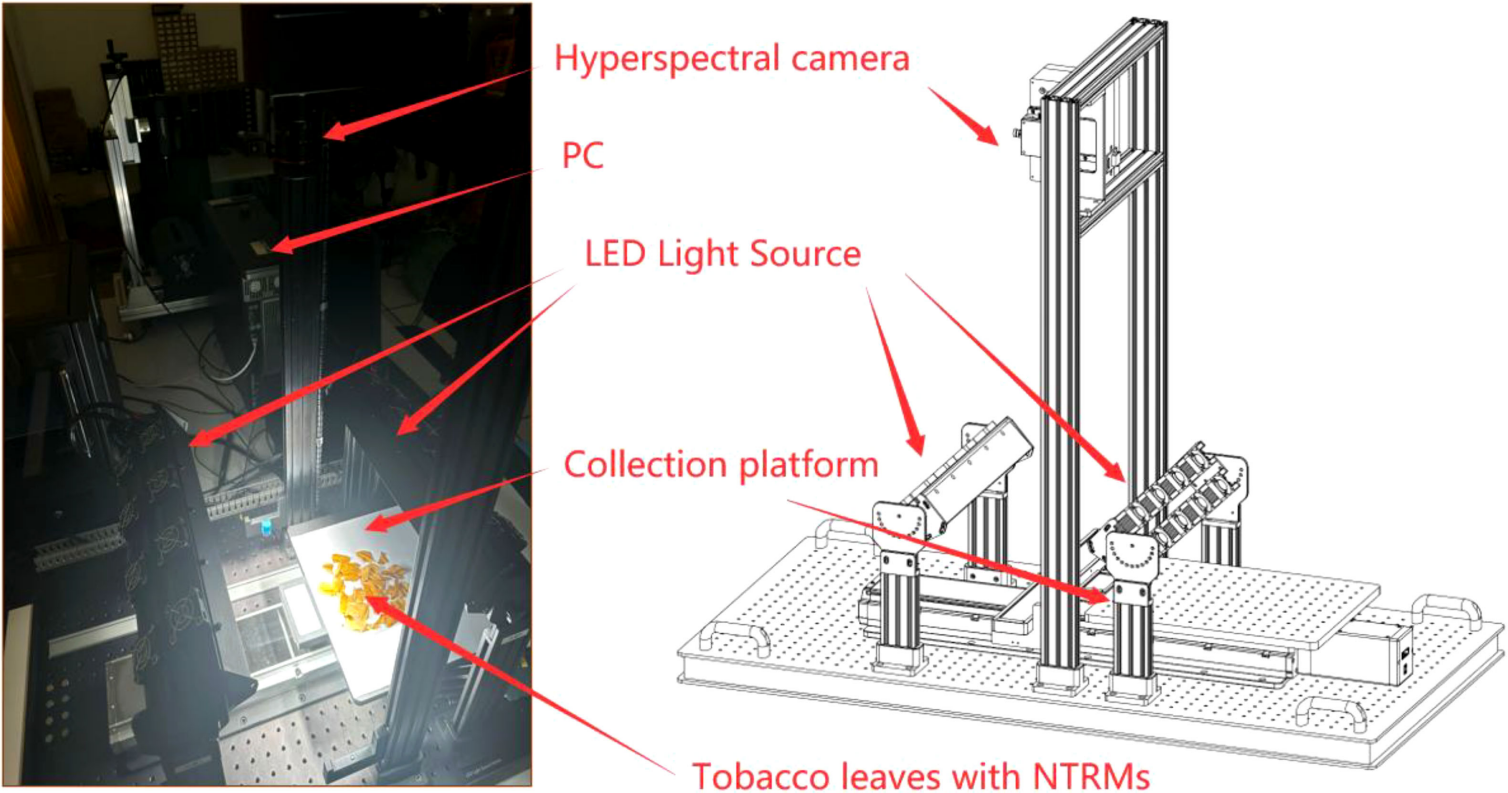
Experimental equipment for simulating the sorting process of tobacco leaves.

The experimental setup for this study is shown in **Figure 2**. The equipment consists of a collection platform, a personal computer (PC), light-emitting diode light (LED light), and a hyperspectral camera, with specific parameters detailed in **Table 1**.

**Table 1:** Main parameters of experimental equipment.

Equipment	Parameter
Hyperspectral Camera	SpectraEye (SEL-24)
Collection platform	1200×600×1050mm
PC	CPU: Intel(R) Xeon(R) Silver 4210R GPU: NVIDIA T1000

### 2.2 Image acquisition

**Figure 3:**
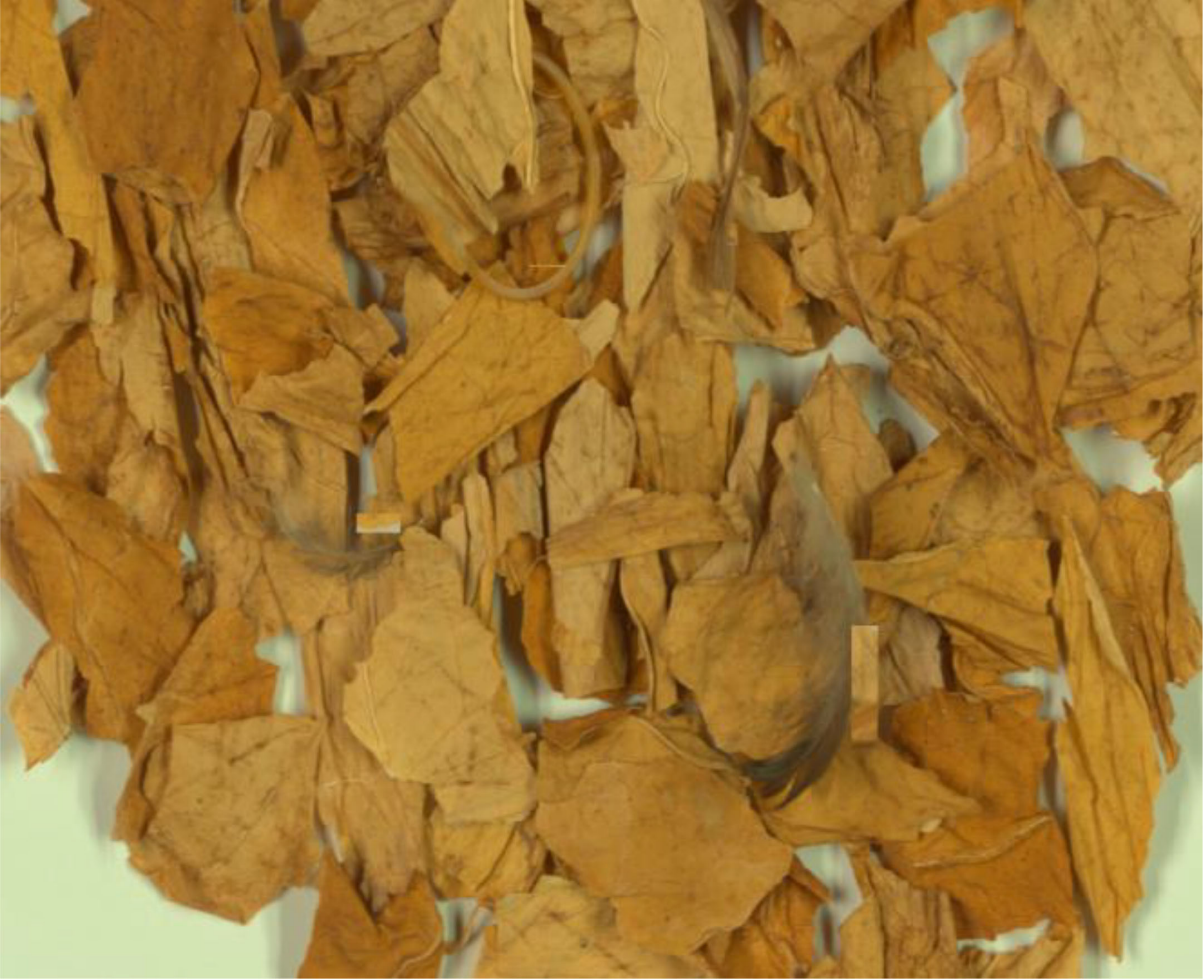
RGB image samples of the collected hyperspectral images.

Tobacco leaves used in this study were planted in various cities in Yunnan Province, China (north latitude: 25°02′58′′, east longitude: 102°42′32′′), and included various varieties of tobacco leaves, such as Yunyan 87, K326, Yunyan 97, etc. The adaptation ability of the model to different weather conditions was improved by adjusting the intensity of the light source. Weeds, rubber rings, and feathers are some of the NTRMs explored. Tobacco leaves and NTRMs were placed first, and photos were taken with the hyperspectral camera positioned directly overhead at the optimal brightness and acquisition rates. We captured images at different transmission speeds, allowing the model to address the issue of motion blur induced by the operational speed of faster transmission devices. Finally, materials were collected at the end. **Table 2** contains details about the HSIs. The study collected 1,000 HSIs with 4,203 NTRMs, taking into account the huge quantity, disordered arrangement, and stacking of tobacco leaves. **Figure 3** shows RGB image samples from the gathered hyperspectral images.

**Table 2:** Main parameters of HSIs.

Parameter	Value
Resolution	3024×2464
Wavelength coverage	400-900nm
Spectral resolution Spectral bands	5nm 101

### 2.3 Spectral analysis

#### 2.3.1 PCA based on different spectral regions

**Figure 4:**
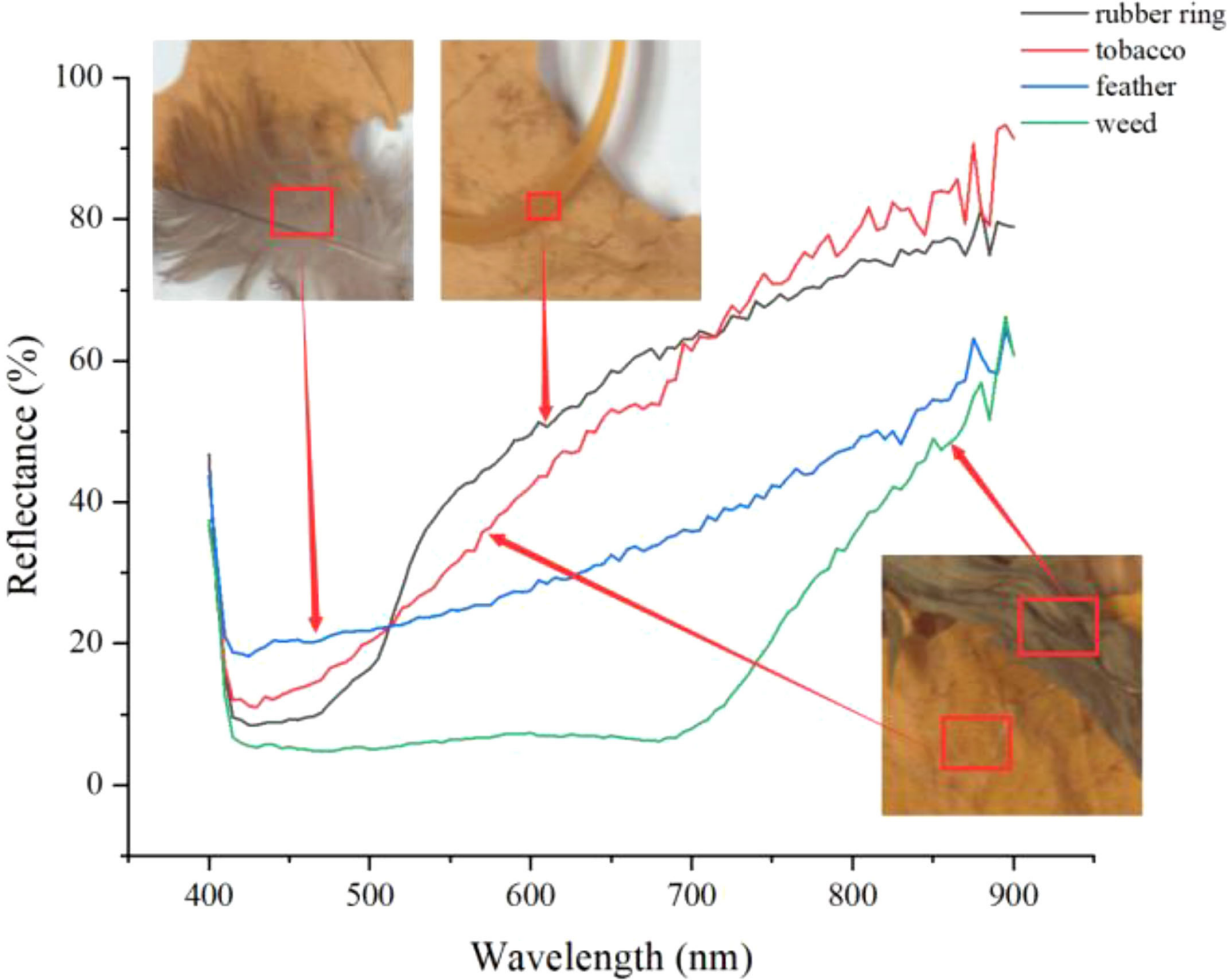
Reflectance curves of the ROIs.

The study used ENVI 6.0 (The Environment for Visualizing pictures, version 6.0) software to perform spectrum analysis on hyperspectral images, as well as Python3.9 and PyCharm 2021.3.1 software was used to assist with data processing. Initially, ROIs for tobacco leaves and NTRMs were extracted from the collected HSIs, with each ROI containing between 8,000 and 30,000 pixels. These ROIs will be used for further spectral analysis in later stages. We will use these ROIs for further spectral analysis. As shown in **Figure 4**, we used the spline regression method for curve smoothing, the reflectance differences between tobacco leaves and NTRMs are more pronounced and the curves are relatively smooth in the wavelength regions around 450 nm and 550-850 nm. For further analysis, the study selected the 550-850 nm and 450-850 nm ranges.

**Figure 5:**
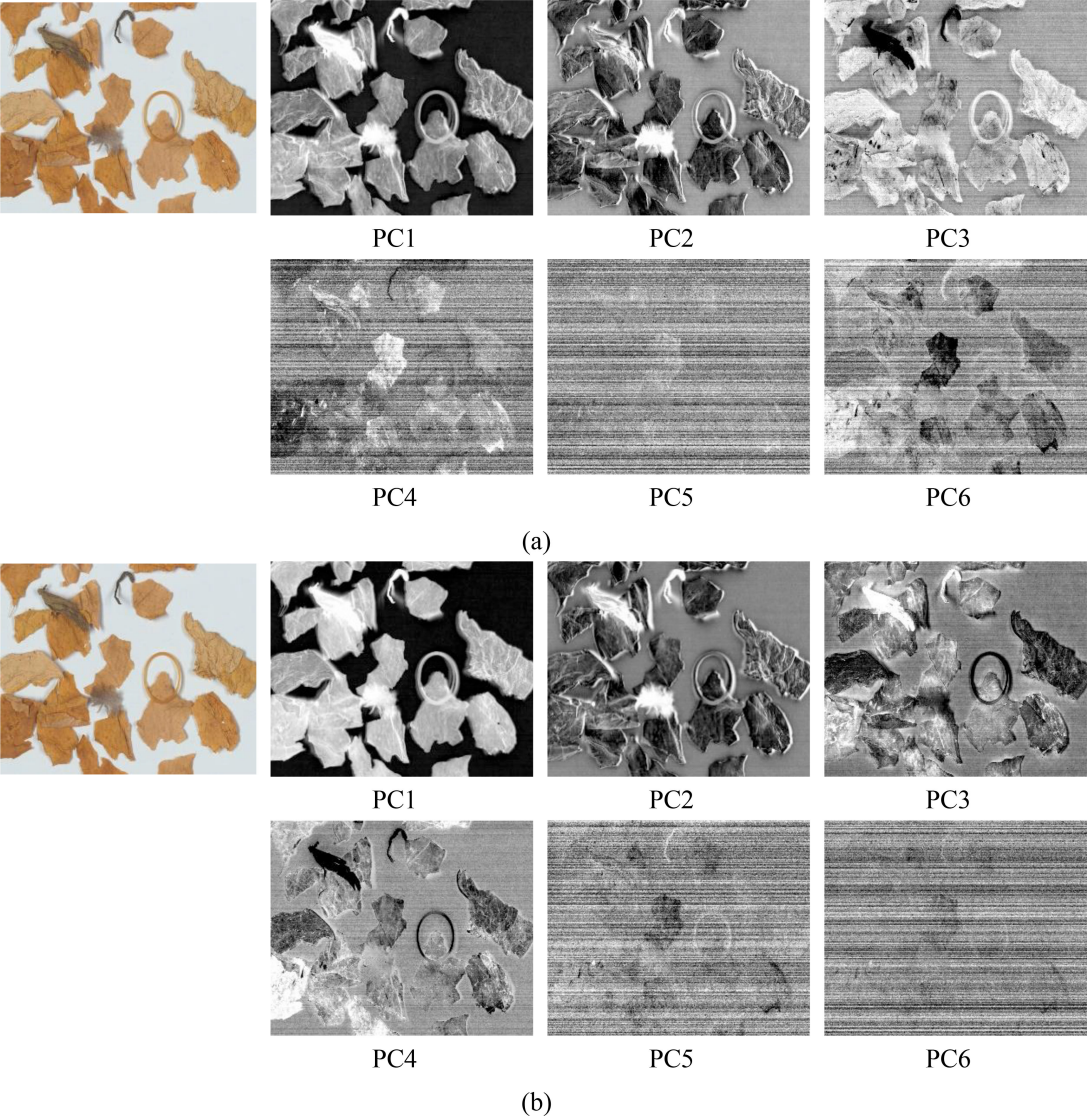
PC score images in the wavelength range: **(a)** 550-850nm, **(b)** 450-850nm.

However, there is significant redundancy among the spectral bands in these data, and performing target detection directly would considerably increase computational load and complexity. Thus, dimensionality reduction of the spectral data is necessary. PCA, an effective dimensionality reduction method (Prasad and Bruce, 2008), is frequently used in HSIs processing (Frederick et al., 2023; Huang et al., 2015; Luo et al., 2019). PCA was performed on these two spectral regions separately, and the resulting PC score images are shown in **Figure 5**. The later-arranged PC images provide less to the hyperspectral data in these two spectral regions because of the decreasing order of variance. Only the first six PC images are shown. In contrast, we can see that the PC1, PC2, and PC3 images in the 550–850nm region and the PC1 and PC2 images in the 450–850nm region show ghosting effects between the shadows of weeds and rubber bands. The PC4, PC5, and PC6 images in the 550-850nm region and the PC5 and PC6 images in the 450-850nm region contain excessive noise. The PC3 image in the 450-850nm region shows better feature representation, but the PC4 image in the same region captures subtle contour changes more effectively, clearly distinguishing object edges and demonstrating stronger NTRMs detection capability. Thus, for further analysis, we chose the PC4 image in the 450–850nm range.

#### 2.3.2 Selection of characteristic wavelengths

**Figure 6:**
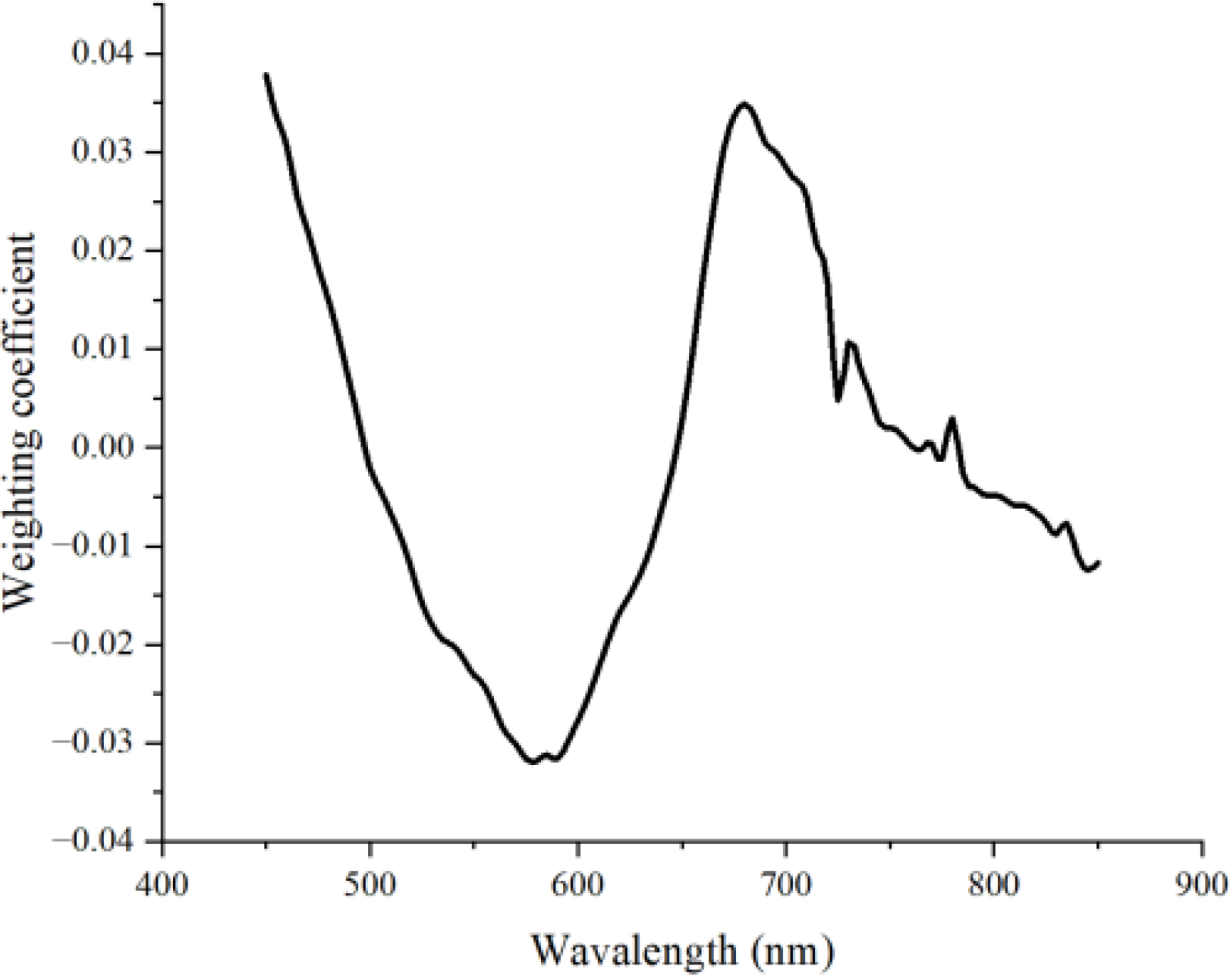
Weight coefficient image of PC4 in the 450-850nm range.


The study will use the PC4 image from the 450-850nm region to select wavelengths that approximately represent the characteristics of the entire region. The weight coefficient image (**Figure 6**) for PC4 can be obtained using Equation 1.

(1) $$\begin{array}{l} \gamma =(\gamma_{1},\gamma_{2},\cdots ,\gamma_{m})=(\frac{\beta_{1,i}}{\sqrt{\alpha_{1,i}}},\frac{\beta_{2,i}}{\sqrt{\alpha_{2,i}}},\cdots ,\frac{\beta_{m,i}}{\sqrt{\alpha_{m,i}}})\\ =[\begin{array}{ccc} \begin{array}{cc} \frac{\beta_{1,1}}{\sqrt{\alpha_{1,1}}} & \frac{\beta_{2,1}}{\sqrt{\alpha_{2,1}}}\\ \frac{\beta_{1,2}}{\sqrt{\alpha_{1,2}}} & \frac{\beta_{2,2}}{\sqrt{\alpha_{2,2}}} \end{array} & \cdots & \begin{array}{c} \frac{\beta_{m,1}}{\sqrt{\alpha_{m,1}}}\\ \frac{\beta_{m,2}}{\sqrt{\alpha_{m,2}}} \end{array}\\ \vdots & \ddots & \vdots \\ \begin{array}{cc} \frac{\beta_{1,n}}{\sqrt{\alpha_{1,n}}} & \frac{\beta_{2,n}}{\sqrt{\alpha_{2,n}}} \end{array} & \cdots & \frac{\beta_{m,n}}{\sqrt{\alpha_{m,n}}} \end{array}] \end{array}$$

where, i∈{1,2,⋯,n} and j∈{1,2,⋯,m} . $\alpha_{i,j}$ . represents the eigenvalue of the principal component i at the wavelength j , $\beta_{i,j}$ denotes the corresponding eigenvector, and $\gamma_{i,j}$ indicates the corresponding weight coefficient.

Based on the correlation coefficient curve, three wavelengths with wide intervals located at peaks and troughs are selected: 580 nm, 680 nm, and 850nm.

**Figure 7:**
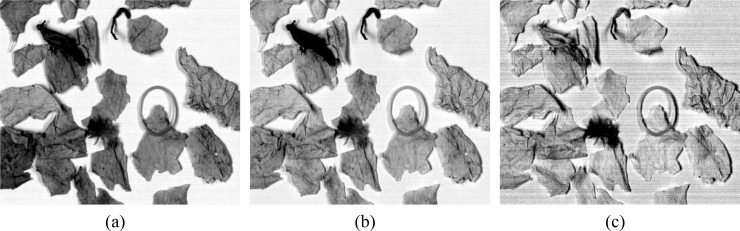
Grayscale images corresponding to different wavelengths: **(a)** 580nm, **(b)** 680nm, **(c)** 850nm.

The chosen three bands were converted into single-wavelength grayscale images, as shown in **Figure 7**, where NTRMs are clearly visible. The contour information of NTRM is mainly reflected in the grayscale image at 580 nm, the contrast between NTRMs and tobacco leaves is enhanced in the grayscale image at 680 nm, and the internal information of NTRMs is primarily reflected in the grayscale image at 850 nm. This suggests that the three bands that were chosen are trustworthy. For the sake of additional study, the complete spectral region will be represented by these three distinctive bands.

### 2.4 Decorrelation contrast stretching

**Figure 8:**
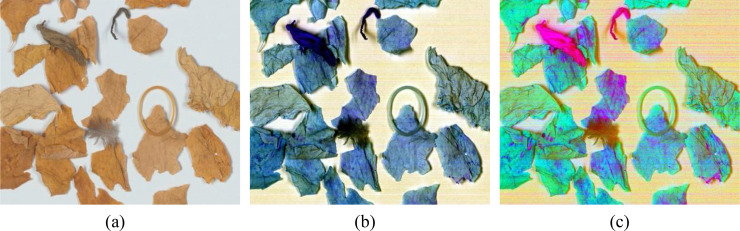
Result images after processing: **(a)** RGB, **(b)** Pseudo color composition (PCC), **(c)** Decorrelation contrast stretching (DCS).

Decorrelation contrast stretching is one of the effective methods for eliminating strong correlations in images (Gillespie, 2003; Gillespie et al., 2003). In multispectral images, it enhances the color difference, resulting in higher color contrast, highlighting complex details, and producing a richer color composite image. Decorrelation stretching is widely used in hyperspectral data (Domingo et al., 2015; Palomar-Vazquez et al., 2017) and has been shown to be very effective in emphasizing subtle details in images (Le Quellec et al., 2015; Rogerio-Candelera, 2015). **Figure 8** shows the pseudo color image fusion and decorrelation stretching applied to the grayscale images of the three selected characteristic wavelengths. PCC and DCS images are the terms used to describe the final images. Following the application of these two processes, it is clear that the NTRMs can be identified. The efficacy of the technique is demonstrated by the DCS image, which displays very obvious contours and color distinctions, even while some weeds and tobacco leaves have similar colors in the RGB image. However, tobacco leaves can also exhibit weed-like colors because of the presence of mold or roots. As a result, deep learning techniques should be used to capture more intricate details like texture and shape.

### 2.5 Dataset construction

**Figure 9:**
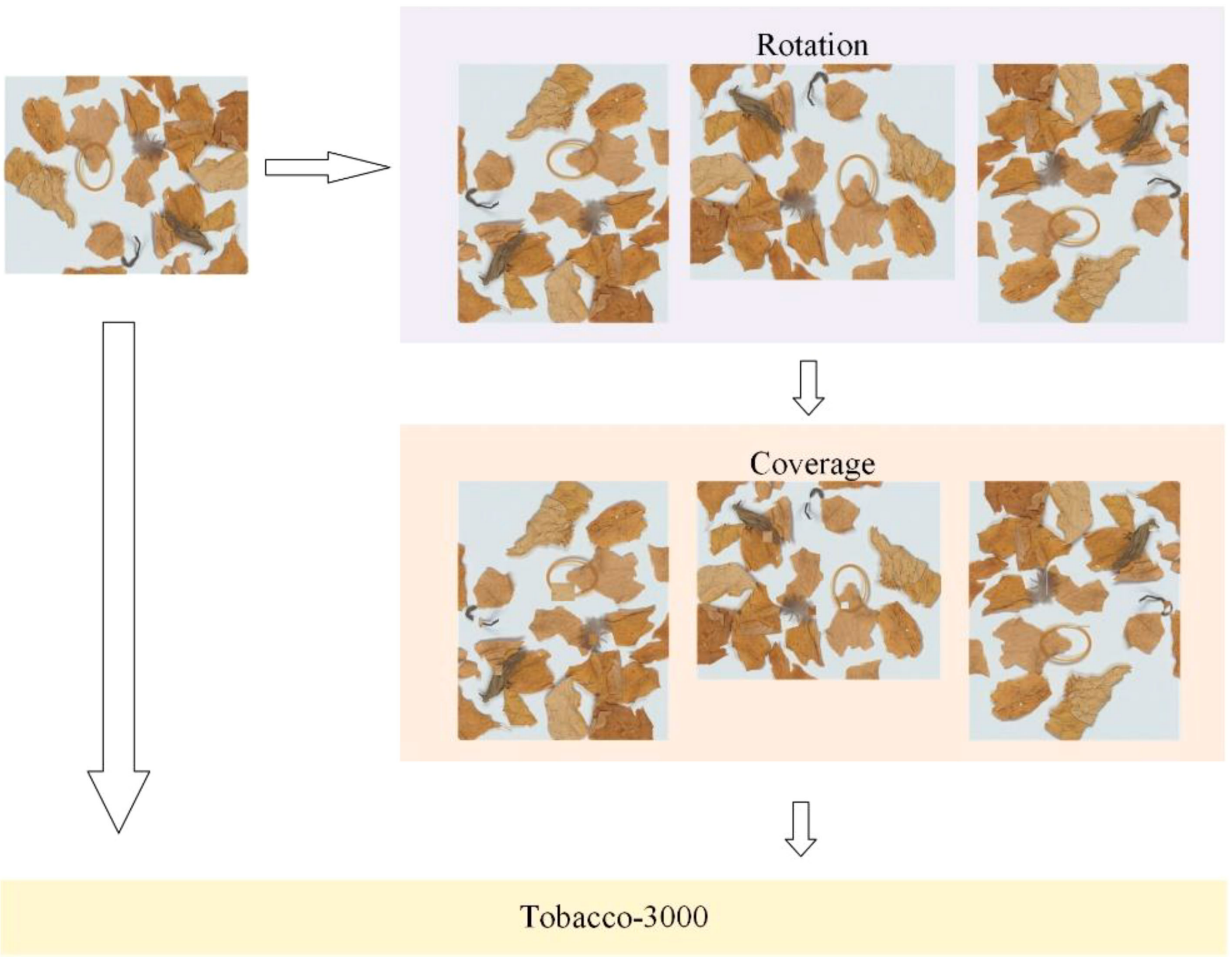
Data augmentation process.

The purpose of data augmentation is to expand the dataset to improve the model’s fitting capability, including methods such as image rotation, cropping, and brightness adjustment (Maharana et al., 2022; Yuan et al., 2023). Only 1000 hyperspectral data samples were gathered for this study because hyperspectral data acquisition, processing, and storage are time-consuming and complex. Image rotation and background filling techniques were selected for data augmentation in order to improve the detection performance of NTRMs under tobacco leaf occlusion. Before data augmentation, the original images were split into training, validation, and test sets in a 6:2:2 ratio, and NTRMs were annotated using the LabelImg tool (https://github.com/HumanSignal/labelImg). The images in these three sets were then performed to data augmentation. As shown in **Figure 9**, each original image was rotated clockwise by 90°, 180°, and 270°, and backgrounds without NTRMs were cut and overlaid onto areas with NTRMs. Three augmented images were created from each original image, and any two of these images were then joined with the original to create the Tobacco-3000 dataset, which had 3000 images with 12,609 NTRMs for model training. The quantities of each type of NTRMs are shown in **Table 3**, indicating that the distribution ratio of each NTRMs is roughly the same across the sets. In order to ensure that the two rotated images chosen for each original image were consistent and allow for fair comparison in later experiments, the study performed the same partitioning and data augmentation processes to PCC and DCS images.

**Table 3:** Data distribution of NTRMs.

Category of NTRMs	Category of NTRMs	Number
Train set	Weed	2697
	Rubber ring	1944
	Feather	2898
Validation set	Weed	882
	Rubber ring	678
	Feather	951
Test set	Weed	960
	Rubber ring	621
	Feather	978

### 2.6 DBY-Tobacco detection model

#### 2.6.1 Improved DBY -Tobacco network structure

The YOLO series of algorithms has evolved to YOLOv11. However, the YOLOv8 algorithm is highly flexible and stable in actual deployment, and it can handle a variety of hardware platforms, ensuring the hardware compatibility of the model. The YOLOv8 algorithm is divided into five versions based on complexity: YOLOv8n, YOLOv8s, YOLOv8m, YOLOv8l, and YOLOv8x. The model needs a lower model complexity and detection time because it is designed for real-time monitoring. Therefore, the DBY-Tobacco model proposed in this study is an improvement based on YOLOv8n.

The structures of YOLO series networks are composed of a backbone, neck, and head:

1. The backbone is composed of a Convolutional Neural Network (CNN), and its primary task is feature extraction at various levels of image granularity. It is capable of extracting feature maps from input images that contain semantic information about the object’s position, shape, color, and texture.
2. The neck receives the feature maps produced by the backbone and uses a series of feature fusion mechanisms, such as the Feature Pyramid Network (FPN), Path Aggregation Network (PAN), and Bidirectional-FPN (BiFPN) (Mingxing et al., 2020; Wang et al., 2022; Xu et al., 2023), to fuse features at different scales, enabling more effective target detection.
3. The head is the component in the YOLO algorithm responsible for generating detection results. Based on the feature maps passed from the neck, the model predicts the targets, producing both the bounding boxes and the class probabilities for the detected objects.

**Figure 10:**
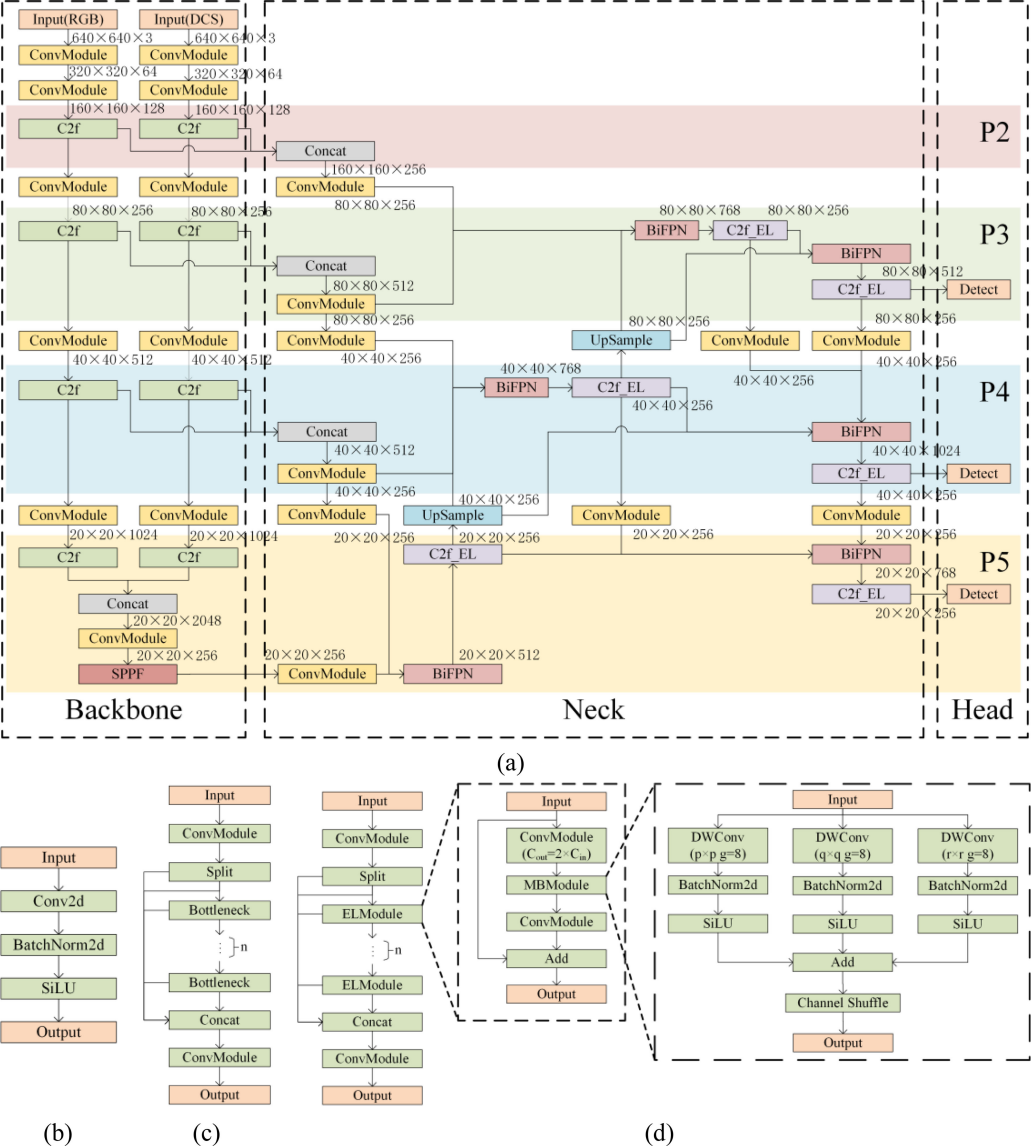
Network architecture of the DBY-Tobacco model and its components: **(a)** DBY-Tobacco, **(b)** ConvModule, **(c)** C2f module, **(d)** C2f_EL module.

The model creates two backbone branches, as illustrated in **Figure 10**, to process RGB and DCS images independently, allowing for the simultaneous extraction of features from each branch’s images. BiFPN-Efficient-Lighting-Feature-Pyramid-Network (BELFPN) is a lightweight and efficient structure that is proposed for more effective feature fusion at the neck part. The primary components of this structure are C2f_Efficient-Lighting (C2f_EL) and BiFPN (Yang et al., 2024). The BiFPN may do multi-scale feature weighted fusion by accepting multiple inputs.

#### 2.6.2 C2f_EL module

The C2f_EL module replaces the Bottleneck in C2f module with the Multi-Branch-Module (ELModule). The ELModule is an efficient and lightweight structure using Multi-Branch-Module (MBModule) as its operator. As shown in **Figure 10d**, the MBModule has three branches that operate in parallel, which speeds up computation. Depthwise Convolution (DWConv) is used in each branch, which significantly reducing the computational load and parameter. Convolutional kernels of various sizes are used by the branches to gather information on multi-scale features, and Channel Shuffle is then used to accomplish multi-scale feature fusion.

While smaller receptive fields are more suited for identifying smaller scale targets, Larger receptive fields are better suited for detecting larger objects (Yanghao et al., 2019). To get multi-scale information, the MBModule chooses different multi-scale convolutional kernels for various feature layers.

#### 2.6.3 Loss function

We used the loss function of the YOLOv8 model, which combines Binary Cross Entropy (BCE) Loss, CIoU Loss, and Distribution Focal Loss (DFL) (Li et al., 2020) with weighted summation. The classification loss is BCE, while the localization losses are CIoU and DFL. The calculation formulas are given in Equations 2–6:


(2) $$L_{BCE}=-(q\log_{2}(p)+(1-q)\log_{2}(1-p))$$

(3) $$DFL(S_{i},S_{i+1})=-((y_{i+1}-y)\log_{2}(S_{i})+(y-y_{i}){\text{log}}_{2}(S_{i+1})$$

(4) $$L_{CIoU}=1-CIoU=1-IoU+\frac{\rho^{2}(b,b^{gt})}{c^{2}}+\alpha v$$

(5) $$\alpha =\frac{v}{1-IoU+v}$$

(6) $$v=\frac{4}{\pi^{2}}{\left(\arctan\frac{w^{gt}}{h^{gt}}-arctan\frac{w}{h}\right)}^{2}$$

where, p represents the probability of a positive prediction, q denotes the label, IoU stands for the Intersection over Union between the predicted box and the ground truth box, y represents the true target value, $y_{i}$ and $y_{i+1}$ are the neighboring boundary values of y , S represents the Softmax function, $\rho^{2}$ denotes the Euclidean distance between two rectangular boxes, b represents the center point of the predicted box, $b^{gt}$ represents the center point of the ground truth box, c is the diagonal distance of the smallest enclosing area containing both rectangles, α is the weight coefficient representing the aspect ratio consistency factor between the predicted box and the ground truth box, v is a balance ratio used to measure the consistency of the relative proportion between two rectangles, w and h denote the width and height of the predicted box, and $w^{gt}$ and $h^{gt}$ represent the width and height of the ground truth box.

## 3 Results and discussions

### 3.1 Environmental configuration

The following hardware platform was used for the experiments: Intel(R) Xeon(R) Platinum 8383C CPU @ 2.70GHz, NVIDIA GeForce RTX 4090. Anaconda3 was used to create the training virtual environment, and **Table 4** displays the code execution environment. YOLOv8n was used as the base network model for the experiments. **Table 5** lists the training hyperparameters.

**Table 4:** Parameters of the virtual environment.

Packages	Version
Ultralytics	8.2.79
Python	3.9.19
Torch	1.11.0
Torchvision	0.12.0
Torchaudio	0.11.0
Cuda	11.5
PyCharm	2021.3.1

**Table 5:** Hyperparameter settings for network training.

Parameters	Values
Batch size	16
Pre-trained weight	False
Epoch	300
Patience	50
Workers	4
Input size	640

### 3.2 Performance evaluation

To assess the performance of the improved DBY-Tobacco model based on detection results, metrics such as Precision (P) as per Equation 7, Recall (R) as per Equation 8, Mean Average Precision (mAP) as per Equations 9, 10, F1 Score (F1) as per Equation 11, Frames Per Second (FPS), parameter count, Giga Floating-point Operations Per Second (GFLOPs), and model size were used. Validation experiments were carried out on the test set to ensure accurate evaluation, and 3000 warm-up iterations were performed to assure complete GPU resource use before measuring FPS.


(7) $$P=\frac{TP}{TP+FP}\;$$

(8) $$R=\frac{TP}{TP+FN}\;$$

(9) $$AP=\int_{0}^{1}P(r)dr$$

(10) $$mAP=\frac{\sum_{i=1}^{n}AP_{i}}{n}$$

(11) $$F1=\frac{2\times P\times R}{P+R}$$

where, TP (True Positive) means correctly predicted positive instances, FP (False Positive) represents incorrectly predicted positive instances, FN (False Negative) denotes actual positive instances that were incorrectly predicted as negative, P(r) represents the precision-recall curve, and n denotes the number of categories.

### 3.3 Ablation experiments

Based on YOLOv8n, a series of improvements were implemented in the study. To validate the effectiveness of these improvements, ablation experiments were designed on the Tobacco-3000 dataset, with results shown in **Table 6**.

**Table 6:** Results of ablation experiments.

Method	Input	Model size	Parameters	GFLOPs	FPS	Precise	Recall	mAP@50	mAP@50-95	F1
YOLOv8n(I1)	RGB	6.3	3,011,433	8.1	126	88	82.9	88.2	65.7	85.4
YOLOv8n(I2)	PCC	6.3	3,011,433	8.1	128	90.5	86.7	90	67.5	88.6
YOLOv8n(I3)	DCS	6.3	3,011,433	8.1	122	91.4	84.2	90.2	67.9	87.7
YOLOv8n(I4)	Multi	9	4,362,137	12.5	103	91.7	87.1	92.3	73	89.3
DBY-Tobacco	Multi	8.5	3,733,674	10.7	**151**	**91.8**	**87.6**	**92.8**	**73.7**	**89.7**

Bold values indicates the maximum value of the column.

In this context, “Multi” refers to using RGB and DCS images as separate input branches for the model. Comparing I2 to I1, all performance metrics showed improvement. I3 showed some improvements over I2, but no enhancement in Recall was observed. This could be owing to the presence of roots or mold in the tobacco leaves, which causes the DCS technique to transform these areas into colors similar to weeds, prompting the model to incorrectly categorize them as NTRMs. I4, which included features from RGB and DCS images, resulted in a considerable overall improvement but increased the computing load and inference time. The DBY-Tobacco model, which was based on I4, included the BELFPN module, which improved overall model performance while reducing complexity and inference time. Overall, the ablation experiments show that HSI technology works and validate the development of the DBY-Tobacco model.

### 3.4 Comparison of algorithms

To assess the performance of the DBY-Tobacco model, in **Table 7**, we compared it to YOLO series methods and previously proposed multispectral object detection models.

**Table 7:** Comparison of the DBY-Tobacco model with other models.

Method	Input	Model size	Parameters	GFLOPs	FPS	Precise	Recall	mAP@50	mAP@50-95	F1
YOLOv3-tiny	RGB	24.4	12,133,670	19	303	89.2	84.5	88.3	64.6	86.8
YOLOv3-tiny	DCS	24.4	12,133,670	19	300	90.6	84.8	90.2	65	87.6
YOLOv5n	RGB	5.3	2,509,049	7.2	123	89.4	83.8	88.7	66.5	86.5
YOLOv5n	DCS	5.3	2,509,049	7.2	126	88.9	83	88.6	66	85.8
YOLOv6n	RGB	8.7	4,238,441	11.9	125	87.7	83.5	86.9	66.1	85.5
YOLOv6n	DCS	8.7	4,238,441	11.9	129	89.9	83.4	89.8	68.6	86.5
YOLOv8n	RGB	6.3	3,011,433	8.1	126	88	82.9	88.2	65.7	85.4
YOLOv8n	DCS	6.3	3,011,433	8.1	122	91.4	84.2	90.2	67.9	87.7
YOLOv9t	RGB	4.7	2,005,993	7.9	64	87.8	82.9	87.6	66.3	85.3
YOLOv9t	DCS	4.7	2,005,993	7.9	63	90.6	83.4	90.4	68.4	86.9
YOLOv10n	RGB	5.8	2,708,210	8.4	114	85.5	79.7	85.5	62.9	82.5
YOLOv10n	DCS	5.8	2,708,210	8.4	119	90	80.7	88.6	65.8	85.1
SuperYOLO	Multi	5.5	2,520,225	63	79	91.5	85.7	91.9	73.1	88.5
CFT	Multi	24.7	12,044,313	869.3	51	89.9	82.7	88.6	66.5	86.1
ICAFusion	Multi	7.7	3,662,521	10.4	109	90.3	87.5	91.4	72.1	88.9
DBY-Tobacco	Multi	8.5	3,733,674	10.7	151	**91.8**	**87.6**	**92.8**	**73.7**	**89.7**

Bold values indicates the maximum value of the column.

Through comparison, it can be concluded that, while YOLOv5n exhibits a decrease of 0.1 in mAP@50 and 0.5 in mAP@50-95 on DCS images compared to RGB images, other models perform better on DCS images, with improvements of approximately 2% in mAP@50 and mAP@50-95. For multi-input models, the proposed DBY-Tobacco model outperformed all other models in Precision, Recall, mAP@50, mAP@50-95, and F1 scores of 87.6%, 87.6%, 92.8%, 73.7%, and 89.7%, respectively. DBY-Tobacco has a slightly higher model size and parameter count than the SuperYOLO and ICAFusion models, but it provides faster inference speed, making it more suitable for practical applications. To summarize, DBY-Tobacco, with its low complexity and high performance, is well-suited for NTRMs detection tasks.

### 3.5 Comparison of validation

**Figure 11:**
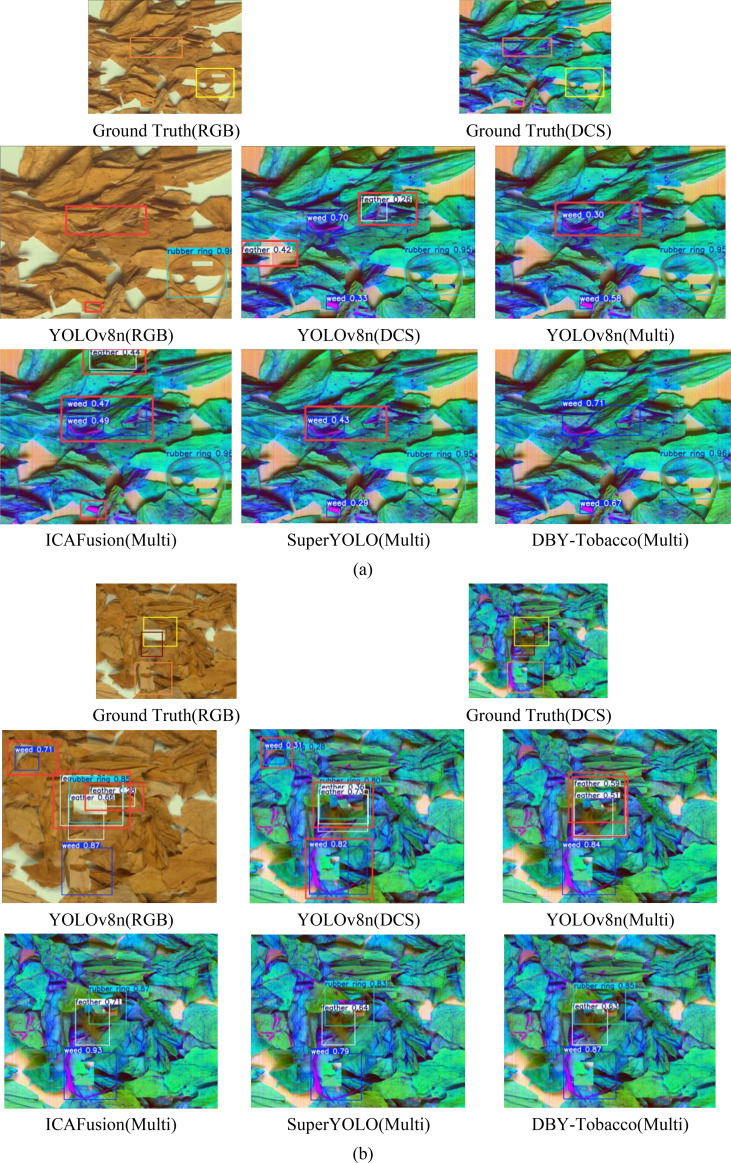
Comparison of different models in practical detection performance: **(a)** Group 1, **(b)** Group 2.

To demonstrate the practical detection capability of the proposed DBY-Tobacco model, it was compared to classic detection algorithms, as shown in **Figure 11**. The orange rectangles in the images represent ground truth boxes for weeds, the yellow rectangles represent ground truth boxes for rubber rings, the brown rectangles represent ground truth boxes for feathers, and the red rectangles represent missed detections, false detections, misdetections, and undetected objects overall.

Through comparison, it is clear that in the RGB images given in **Figure 11a**, the YOLOv8n model missed and incorrectly detected weeds with colors similar to tobacco leaves. This problem improved in DCS images, but the model misidentified certain tobacco leaves and shadows in DCS images as feathers, indicating that the model needs to learn more detailed information. However, by combining RGB and DCS images, these issues were considerably reduced. The YOLOv8n model did not perform well in detecting overlapping NTRMs due to tobacco leaf accumulation and blockage between NTRMs, as shown in **Figure 11b**. However, the ICAFusion model (Shen et al., 2024), SuperYOLO model (Zhang et al., 2023), and DBY-Tobacco model performed better in detecting these NTRMs. Further comparison shows that in **Figure 11a**, the DBY-Tobacco model has more accurate bounding boxes and better confidence scores. The analysis presented above confirms the advanced nature of both hyperspectral technology and the proposed DBY-Tobacco model.

## 4 Conclusion

This study effectively detected NTRMs using HSI and computer vision technologies. We collected 1,000 HSIs in the spectral region from 400nm to 900nm and used PCA to reduce dimensionality, allowing us to select three wavelengths (580nm, 680nm, and 850nm). The image was processed further using pseudo color composition and decorrelation contrast stretch. In order to improve the robustness and generalization ability of the model, we performed data augmentation. To improve model performance, DBY-Tobacco model was proposed, which including a dual-branch backbone and a novel BELFPN module. The DBY-Tobacco achieved F1, mAP@50, mAP@50-95, recall, precision, and FPS scores of 89.7%, 92.8%, 73.7%, 87.6%, 91.8%, and 151, respectively. These results demonstrated the satisfactory robustness and applicability of the DBY-Tobacco model. It is worth noting that the DBY-Tobacco model has great potential for practical applications and can be applied to a variety of multispectral target detection tasks.

The DBY-Tobacco model proposed in this paper achieved a processing speed of 151 FPS in the test, which is significantly faster than most YOLO series target detection algorithms, indicating that it can process hyperspectral images in real time and is suitable for dynamically changing production environments. During deployment, the model can dynamically adapt to different processing rates. More specifically, the model can maintain efficient operation at different production rates by adjusting the processing window (such as batch size or inference time interval) based on the actual needs of the production line. To further increase the calculation speed of the model, we will use pruning technology to reduce the number of parameters and calculation amount of the model to achieve lightweight model, and improve the detection speed through parallelization and distributed processing technology. Besides, long time, high frequency real-time reasoning can lead to high power consumption, affecting the stability of the entire system, but also increase long-term operating costs. Therefore, we will optimize the model for hardware adaptation to run on NVIDIA Jetson to reduce power consumption and improve compute efficiency to increase productivity. Besides, to improve research accessibility, we will develop a software platform that is compatible with existing tobacco production equipment and management systems, and an intuitive graphical user interface will be designed, allowing operators to acquire and process hyperspectral pictures using a simple interface without complex processes.

However, there are still some limitations in the study. For example, HSI technology can introduce stripe noise (Dai et al., 2024; Wang, 2016), which impacts detection tasks. Removing this noise will be a key consideration in future study. Furthermore, the study only examined feathers, rubber rings, and weeds. Other forms of NTRMs (such as cloth strips and glass) are yet to be researched. Future studies will collect images containing a wider variety of NTRMs in order to improve tobacco product quality. At the same time, consider that acquiring and processing hyperspectral images is a complex and resource-intensive task. Future work will combine cutting-edge artificial intelligence approaches, including diffusion models, HSIs generation models, snapshot hyperspectral imaging, and hyperspectral image super-resolution frameworks (Lai et al., 2024; Pang et al., 2024), to enrich the dataset and ensure that downstream activities go smoothly.

In this study, we used PCA to extract the characteristic wavelengths of NTRMs for detection, selecting three representative bands (580 nm, 680 nm, and 850 nm) to allow for lightweight computing of the model. However, this method does not use the entire spectrum curve information and ignores other crucial information, such as inter-band correlations within the spectral sequence. In some complex scenarios, such as when target features are confused with the background, relying just on the chosen bands may be insufficient. The spectral curve contains rich continuous spectral information that can help distinguish between hard-to-differentiate targets and backgrounds. Therefore, in future work, we will consider incorporating the spectral curve data into deep learning networks to accomplish combined learning of spectral and spatial features. We intend to include innovative advances in state-space models and recurrent neural networks (Yao et al., 2024), with the goal of lowering the computing scale of hyperspectral data while maintaining both short-term and long-term contextual relationships between spectral bands.

## Data Availability

The datasets presented in this study can be found in online repositories. The names of the repository/repositories and accession number(s) can be found in the article/supplementary material.
